# Native mitral valve infective endocarditis caused by *Staphylococcus warneri*: A case‐based review

**DOI:** 10.1002/ccr3.4476

**Published:** 2021-07-21

**Authors:** Ibuki Kurihara, Katsuyuki Yoshida, Takahiko Fukuchi, Hitoshi Sugawara

**Affiliations:** ^1^ Division of General Medicine Department of Comprehensive Medicine 1 Saitama Medical Center Jichi Medical University Saitama‐shi Japan

**Keywords:** cardiovascular disorders, geriatric medicine, infectious diseases

## Abstract

In the era of a severely aging population, physicians should pay attention to look for both infective endocarditis and disseminated lesions when blood cultures reveal *Staphylococcus* *warneri*, especially in elderly people with valvular heart disease.

## Graphical abstract

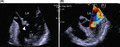

## 1 INTRODUCTION

Studies reporting *Staphylococcus* *warneri* in infective endocarditis (IE) are rare. We presented a 72‐year‐old woman with native mitral valve *S*.* warneri* IE associated with spondylitis and cerebellar infarction. Physicians should be wary of IE and disseminated lesions when blood cultures reveal *S*.* warneri*, especially in elderlies with valvular heart disease.

*Staphylococcus warneri* is a type of coagulase‐negative staphylococci (CoNS) and is a part of the normal flora of the skin, especially the nares, head, legs, and arms.^1^ *Staphylococcus* *warneri* is present in about 50% of healthy adults and represents approximately 4.0%–7.8% of all skin staphylococci in healthy adults.^2^, ^3^, ^4^, ^5^, ^6^, ^7^, ^8^, ^9^ *Staphylococcus* *warneri* is not frequently recognized as a significant human pathogen but is occasionally isolated from immunocompromised patients or patients with medical prosthesis, such as prosthetic heart valves, central venous catheter, and disk prosthesis.^1^ *Staphylococcus* *warneri* is rarely reported as a causative agent of infective endocarditis (IE).^1^ Here, we reported a rare case of native mitral valve IE caused by *S*.* warneri* in a 72‐year‐old Asian woman who had mitral valve regurgitation without valvular heart prosthesis. To analyze the demographic characteristics, predisposing factors, comorbidities, and outcomes of such cases, we reviewed previously reported cases of IE caused by *S*.* warneri*.

## 2 CASE HISTORY

**FIGURE 1:**
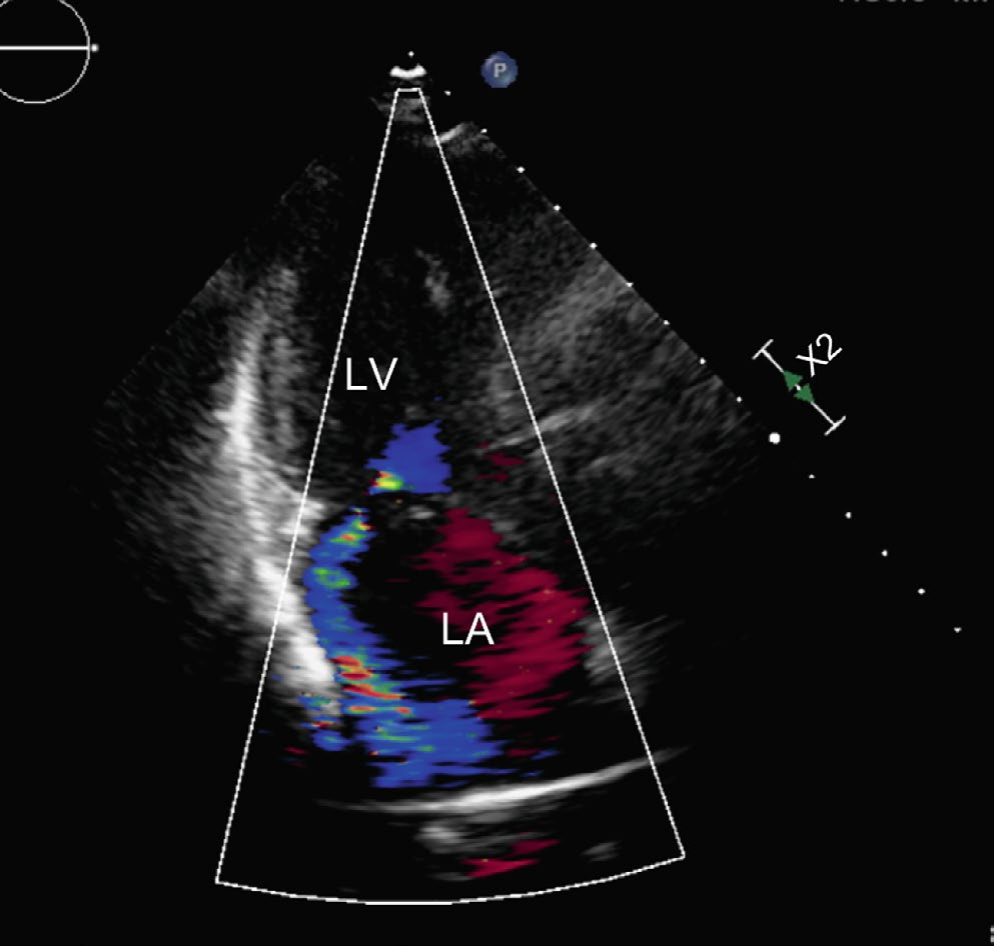
Transthoracic echocardiography findings 3 months before admission. There is moderate mitral valve regurgitation and no vegetation on any valves

A 72‐year‐old Asian woman was admitted to our hospital with a 3‐week history of intermittent fever and general fatigue. Her past medical history was notable for moderate mitral valve regurgitation, which was stable and being followed by a cardiovascular surgeon every year with no medication for 10 years. She has undergone total hysterectomy for uterine myoma at the age of 47 years. Three months before admission, transthoracic echocardiography showed moderate mitral valve regurgitation (Figure 1) and no vegetation on any valves. She had no recent medical histories of diabetes mellitus, weight loss, odontotherapy, and skin disease. Moreover, she did not smoke or drink.

Physical examination showed body mass index of 19.3 kg/m^2^, body temperature of 38.4°C, regular heart rate at 100 beats/min, blood pressure of 95/54 mmHg, respiratory rate of 12 breaths/min, and oxygen saturation of 98% on room air. She was known to have a grade 2 or greater pansystolic murmur at the apex, a Janeway lesion on the sole of the left foot, and no skin lesions. The remainder of the examination, including the range of motion of lumbars and neurologic examination, was unremarkable.

**FIGURE 2:**
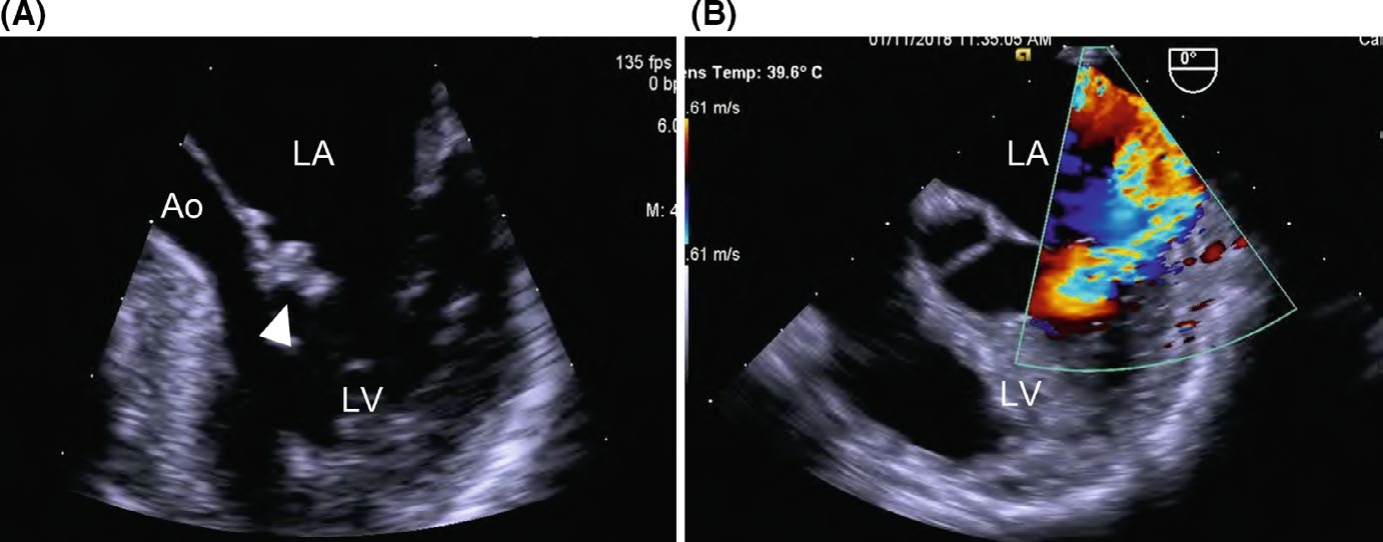
Transesophageal echocardiography on admission. There is a 5‐mm motile vegetation (arrow head) on the anterior cusp of the mitral valve (A) and mitral valve regurgitation (B)

**FIGURE 3:**
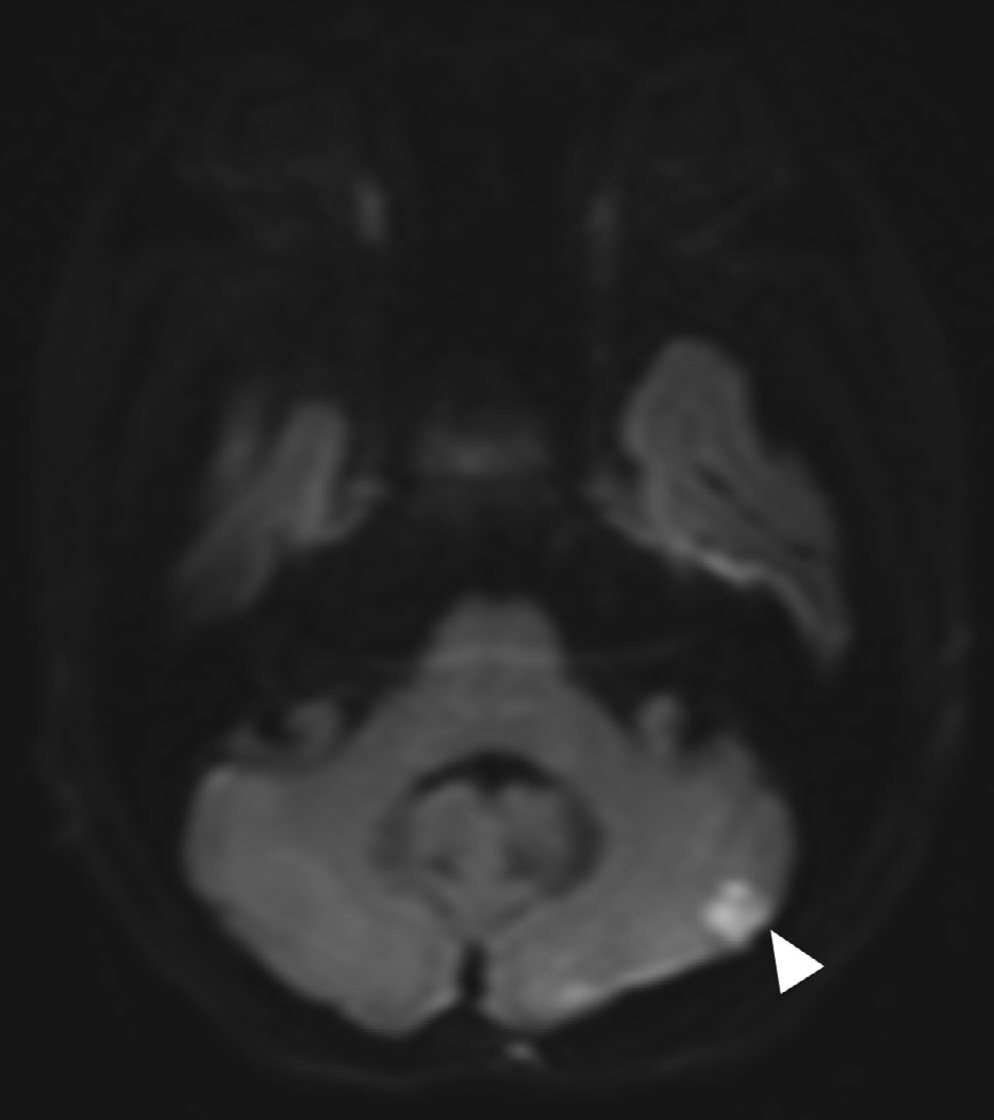
Magnetic resonance imaging of the brain. On diffusion‐weighted images, there is a high‐signal area on the left cerebellum (arrow head)

**FIGURE 4:**
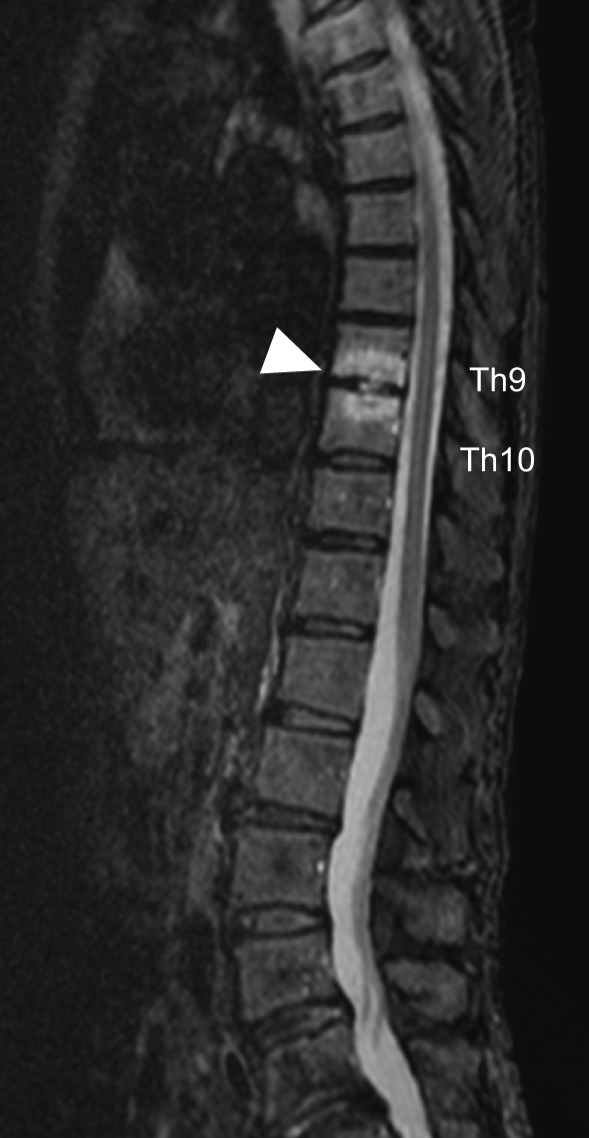
Magnetic resonance imaging of the spine. Short T1 inversion recovery reveals a high‐signal area in the T9–10 disk and T9–10 vertebral body (arrow head)

White blood cell count was 5530/µl, hemoglobin was 10.3 g/dl, C‐reactive protein (CRP) level was 1.43 mg/dl, and erythrocyte sedimentation rate (ESR) was 78 mm/h. All four sets of blood cultures revealed *S*. *warneri*. Transesophageal echocardiography showed a 5‐mm motile vegetation on the anterior cusp of the mitral valve (Figure 2A,B) and mitral valve regurgitation. Contrast‐enhanced thoracic and abdominal computed tomography showed no abscess. Although the patient did not have any neurological symptoms or lumbago, we performed brain and spinal magnetic resonance imaging (MRI) to check for disseminated lesions in infective endocarditis according to the ESC Guidelines for the management of infective endocarditis.^10^ Brain diffusion‐weighted MRI revealed a high‐signal area on the left cerebellum (Figure 3). Sagittal short‐T1 inversion recovery MRI demonstrated the high‐signal lesions on the disk between the 9th and 10th thoracic vertebrae and vertebral bodies (Figure 4). These findings met the two major and three minor Duke criteria for a definitive diagnosis of IE.^11^

She was treated with initially cefazoline of 2 g every 8 h. Based on the subsequent antimicrobial susceptibility test results, we changed the antibiotics to intravenous penicillin G four million units every 4 h for 6 weeks, after the blood culture turned out negative on day 3. On day 14, transesophageal echocardiography showed resolution of the vegetation. She was discharged on day 45 and was continued on treatment with oral amoxicillin 250 mg every 8 h for 6 months, until the CRP and ESR normalized.^12^

## 3 DISCUSSION

This report highlighted the fact that *S*. *warneri*, which has not been recognized frequently as a significant human pathogen, caused IE in an elderly patient who had mitral valve regurgitation, which had been followed up conservatively for 25 years without medical prosthesis. Furthermore, the *S*.* warneri* IE ran a severe course, such as development of disseminated lesions in the skin, brain, and spine.

Two important clinical issues arose from the clinical course presented here. First, *S*. *warneri* can cause IE in a patient with valvular heart disease without medical prosthesis. Second, *S*.* warneri* IE can run a severe course, such as development of disseminated lesions, in an elderly patient.

First, *S*.* warneri* can cause IE in a patient with valvular heart disease without medical prosthesis. To our best of knowledge, there were only 12 case reports, including our case, on IE caused by *S*.* warneri* in the English language literature (Table 1).^13^, ^14^, ^15^, ^16^, ^17^, ^18^, ^19^, ^20^, ^21^, ^22^ The cases had a mean age of 56 years (range, 32–79 years), and 3 of 12 cases were women. In Table 1, 7 of 12 patients (58.3%) without medical prosthesis developed IE. Of these patients, three were immunocompromised because of liver cirrhosis, renal cell carcinoma, and type 1 diabetes mellitus. Two of the three immunocompromised patients underwent skin incision, which could have been one of the risk factors for *S*.* warneri* IE. The present patient did not have any medical prosthesis or skin incision. Instead, mitral valve regurgitation was considered to have predisposed the patient to develop native valve endocarditis (NVE). Review of CoNS NVE cases showed an incidence of 34% among the cases that had valvular heart disease.^23^ Elderly people have been pointed to be more likely to have degenerative valvular heart diseases.^24^ Moreover, patients with valvular heart disease had been discussed to be at risk of developing NVE caused by CoNS, including *S*.* warneri*. Detection of *S*.* warneri* in the blood culture of patients with valvular heart disease of any kind should not be merely recognized as contamination, and physicians should pay attention to the development of IE.

**TABLE 1:** Case reports of infective endocarditis caused by *Staphylococcus warneri*

Pt	Reference	Age (years)	Sex	Medical prosthesis	Skin incision before onset	Comorbidities	Disseminated lesions	Valve	Treatment and outcome (one dead case)
1	Dan et al.^13^	32	M	None	(+)	Vasectomy (2 weeks before)	Embolism popliteal artery	A (Native)	AVR Penicillin + Gentamycin 4 weeks
2	Kamath et al.^14^ (autopsy case)	64	M	None	(−)	Cirrhosis of liver	Splenic infarcts Septic renal emboli	M, A, Pulmonary (Native)	Antibiotics 2 weeks (dead case)
3	Kini et al.^15^	78	F	None	(−)	Atrial fibrillation, Bilateral heart enlargement	(−)	M (Native)	Nafcillin 6 weeks
4	Bhardwaj et al.^26^	59	M	None	(+)	Renal cell carcinoma (right nephrectomy), Scalp laceration (suturing 2 weeks before)	(−)	M (Native)	Cefazolin 6 weeks
5	Diaconu et al.^16^	79	M	None	(−)	Degenerative aortic valve disease	(−)	A (Native)	Oxacillin 6 weeks
6	Wood et al.^17^	66	M	Disk prosthesis Total hip replacement	(−)	(−)	Spondylitis	A, M (Native)	AVR, MVR Vancomycin + gentamycin 6 weeks
7	Stollberger et al.^18^	48	M	L4‐5‐disk prosthesis	(−)	(−)	(−)	A (Native)	Rifampicin + Fusidic acid
8	Abgrall et al.^19^	71	M	Prosthesis aortic valve	(−)	(−)	(−)	A (Prosthetic)	AVR Vancomycin + Pefloxacine 6 weeks
9	Arslan et al.^20^	43	F	Prosthesis aortic valve, silicon mammoplasty	(−)	(−)	(−)	A (Prosthetic)	Ampicillin + Gentamicin 8 weeks
10	Kuvhenguhwa et al.^21^	67	M	Tissue aortic valve	(−)	(−)	(−)	A (Prosthetic)	Vancomycin + Rifampin 6 weeks
11	Yamamoto et al.^22^	59	M	None	(−)	Type 1 DM, Bicuspid aortic valve	(−)	M and A (Native)	MVR and AVR Cefazolin 4 weeks
12	Our patient	72	F	None	(−)	Degenerative mitral valve disease	Discitis Cerebral septic emboli	M (Native)	Penicillin G 6 weeks

Abbreviations: A, aortic valve; AVR, aortic valve replacement; DM, diabetes mellitus; M, mitral valve; MVR, mitral valve replacement.

Second, *S*. *warneri* IE can run a severe course, such as development of disseminated lesions, in patients without any comorbidity. As shown in Table 1, the mortality rate of *S*.* warneri* IE and NVIE was 8.3% (1 of 12 cases) and 11.1% (1 of 9 cases). In the systematic reviews on CoNS infections, including *S*. *warneri* NVE, the reported mortality rate was 19%–25%.^23^, ^25^, ^27^ The present patient had disseminated lesions, such as left cerebellar infarction, spondylitis, and discitis. Moreover, 4 of 12 cases (33.3%) shown in Table 1 had disseminated lesions. In the systematic reviews on CoNS NVE, the reported incidence rate of disseminated lesions was 22%.^25^

Although *S*. *warneri*, unlike most other CoNS, has not been frequently recognized as a significant human pathogen, the mortality rates for *S*. *warneri* and CoNS are higher than for *Streptococcus viridans*.^25^ The high CoNS NVE mortality has two possible reasons, including the delay in diagnosis and background comorbidities. CoNS, including *S*. *warneri*, are slow‐growing, may lead to an indolent course, and commonly contaminate blood culture,^26^ all of which can lead to delayed diagnosis. Chu et al.^25^ reported that patients with CoNS NVE were more likely older (median age, 68 vs. 59 years) and had more prolonged indwelling intravascular catheter (20.0% vs. 1.0%) or healthcare‐associated IE (40.0% vs. 1.34%) as compared to those with *S*. *viridans* NVE. Table 1 shows that of eight cases (median age, 62 years) with *S*. *warneri* NVE, two (25%) had a medical prosthesis and six (75%) had comorbidities such as liver cirrhosis, renal cell carcinoma, bilateral heart enlargement, disk prothesis, and degenerative aortic valve disease.

In conclusion, we report a case of NVE caused by *S*.* warneri* associated with spondylitis and cerebellar infarction in an elderly patient who had recognized mitral valve regurgitation for many years without heart valvular prosthesis. *S*. *warneri* can cause IE in a patient without medical prosthesis and can run a severe course, such as development of disseminated lesions. When blood cultures reveal *S*. *warneri* in patients with valvular heart diseases, physicians should consider IE. In the era of a severely aging population, physicians should pay attention to look for both IE and disseminated lesions when blood cultures reveal *S*.* warneri*, especially in elderly people with valvular heart disease.

## CONFLICT OF INTEREST

None declared.

## AUTHOR CONTRIBUTION

KY: contributed to this work by obtaining patient consent and management of patient. TF and HS: contributed to this work by writing and reviewing the manuscript.

## Data Availability

All data generated or analyzed during this study are included in this published article and its supplementary information files.
